# Evaluation of a novel micro/nanofluidic chip platform for the detection of influenza A and B virus in patients with influenza-like illness

**DOI:** 10.1186/s13568-019-0791-8

**Published:** 2019-05-28

**Authors:** Runqing Li, Wei Gai, Dong Zhu, Chonghou Lok, Cuidan Song, Jingxiao Dong, Ning Han, Yan Zhang, Xiuying Zhao

**Affiliations:** 1 Beijing Tsinghua Changgung Hospital, School of Clinical Medicine, Tsinghua University, 168 Litang Road, Changping District, Beijing, 102218 People’s Republic of China; 2 National Engineering Research Center for Beijing Biochip Technology, 18 Life Science Parkway, Changping District, Beijing, 102206 People’s Republic of China

**Keywords:** Influenza, Influenza-like illness, Rapid influenza diagnostic test, Nucleic acid sequence-based amplification

## Abstract

We introduced a novel micro/nanofluidic chip platform (MNCP), which is based on an isothermal nucleic acid amplification method. This study aimed to evaluate the MNCP method for influenza A and B viruses detecting and subtyping using throat swab samples from patients with influenza-like illness (ILI). A total of 266 throat swab samples from 266 non-repeated patients with ILI were tested for influenza A and B viruses using three methods, MNCP, a rapid influenza diagnostic test (RIDT), and real-time reverse transcription polymerase chain reaction (rRT-PCR). The results of MNCP were compared to those obtained by rRT-PCR and RIDT and the performance of MNCP was further evaluated. Compared with rRT-PCR results, the rates of sensitivity, specificity, overall concordance, and the kappa value of MNCP were 98.89%, 96.97%, 97.65%, and 0.95 for influenza A virus; 94.95%, 99.38%, 97.68%, and 0.95 for influenza B virus, respectively. Subtypes of influenza A viruses, e.g., A(H1N1)pdm09, A(H3N2), and A(not subtyped), and influenza B viruses could be distinguished in one MNCP assay within 1 h. Compared with rRT-PCR and MNCP, RIDT showed poor clinical sensitivity for influenza virus detection. This study showed MNCP is rapid, sensitive and versatile detecting system with potential for clinical application in pathogen diagnosis for patients with ILI.

## Introduction

Pathogen diagnosis of influenza-like illness (ILI) has received increasing attention due to outbreaks of influenza and increased threat to human health from avian influenza (Bedford et al. 2015; Li et al. 2014; Zhu et al. 2019). Prompt and accurate detection of influenza virus is essential for patients with ILI, which helps to avoid unnecessary use of antibiotic therapy and to prevent nosocomial transmission of influenza virus (Neuzil et al. 2000; Townsend and Eiland 2006). Here, we introduced a novel micro/nanofluidic chip platform (MNCP) for influenza virus identification and its value in clinical application was evaluated by comparing MNCP with a rapid influenza diagnostic test (RIDT) and real-time reverse transcription polymerase chain reaction (rRT-PCR).

## Materials and methods

### Study design and sample collection

**Fig. 1:**
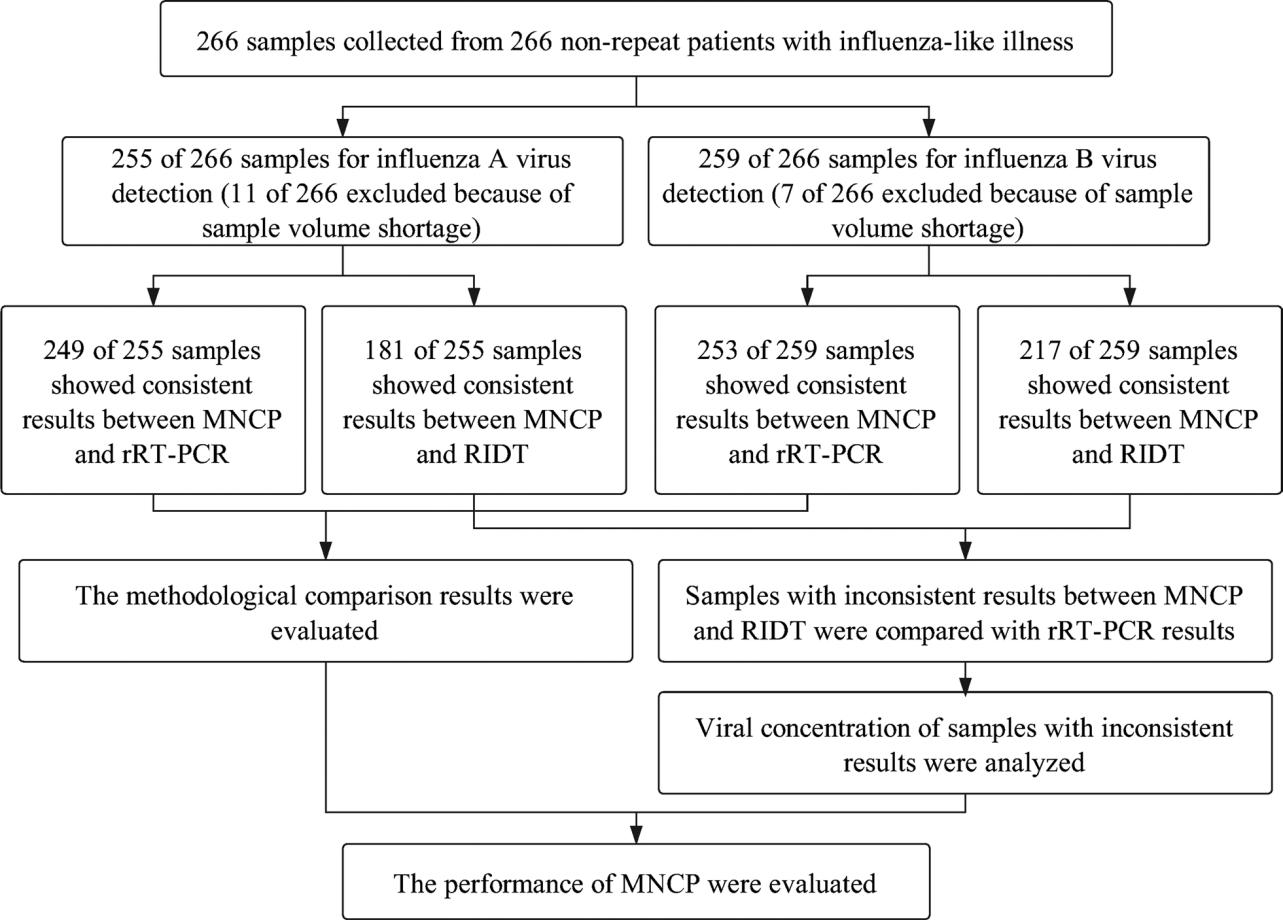
Flowchart of the study. MNCP: micro/nanofluidic chip platform; RIDT: rapid influenza diagnostic test; rRT-PCR: real-time reverse transcription PCR

A total of 266 throat swab samples were collected from 266 non-repeat patients with ILI according to the criteria of diagnostic and treatment protocol for influenza between Dec. 2017 and Feb. 2018 in Beijing Tsinghua Changgung Hospital (Beijing, China). The swab samples were obtained from the surface of the tonsils and the posterior pharyngeal wall by qualified personnel using nylon fiber flocked swabs. Duplicate samples were collected, one was subjected to prompt RIDT assay, the other was immediately placed in a tube containing virus transport medium (Yocon Biological Pharmacy, Beijing, China) and stored at − 80 °C for rRT-PCR and MNCP assays within 3 months (Eisfeld et al. 2014). The study was approved by the ethics committee of Beijing Tsinghua Changgung Hospital (Approval No. 17120-0-01). The flowchart of the study was shown in Fig. 1.

### Design of MNCP

**Fig. 2:**
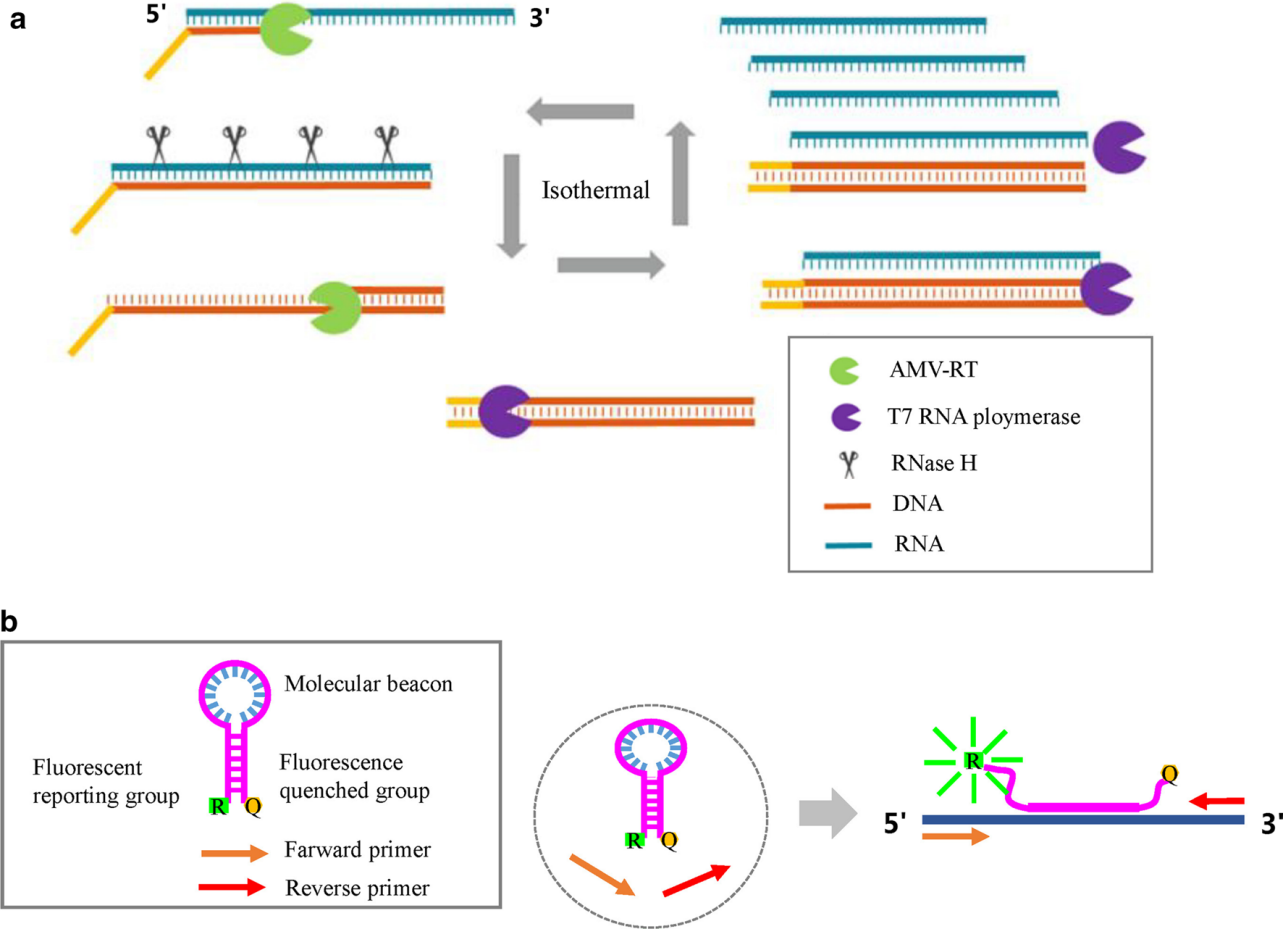
Principles of the MNCP based on NASBA amplification. **a** The isothermal cycle of the NASBA amplification. **b** Signal reporting of the amplification product. AMV-RT: avian myeloblastosis virus reverse transcriptase; MNCP: micro/nanofluidic chip platform; NASBA: nucleic acid sequence-based amplification

MNCP was developed based on the principle of nucleic acid sequence-based amplification (NASBA), which is a homogeneous and isothermal reaction (Compton 1991). In a typical NASBA process, three enzymes, avian myeloblastosis virus reverse transcriptase (AMV-RT), RNase H, and T7 RNA polymerase work together, giving the ability to exponentially amplify single-stranded RNA (Rodríguez-Làzaro et al. 2004) (Fig. 2a). Molecular beacon probes specifically bind to the amplification products, resulting in fluorescence emission that is due to fluorescence resonance energy transfer and can be detected by a detector (Fig. 2b).

**Fig. 3:**
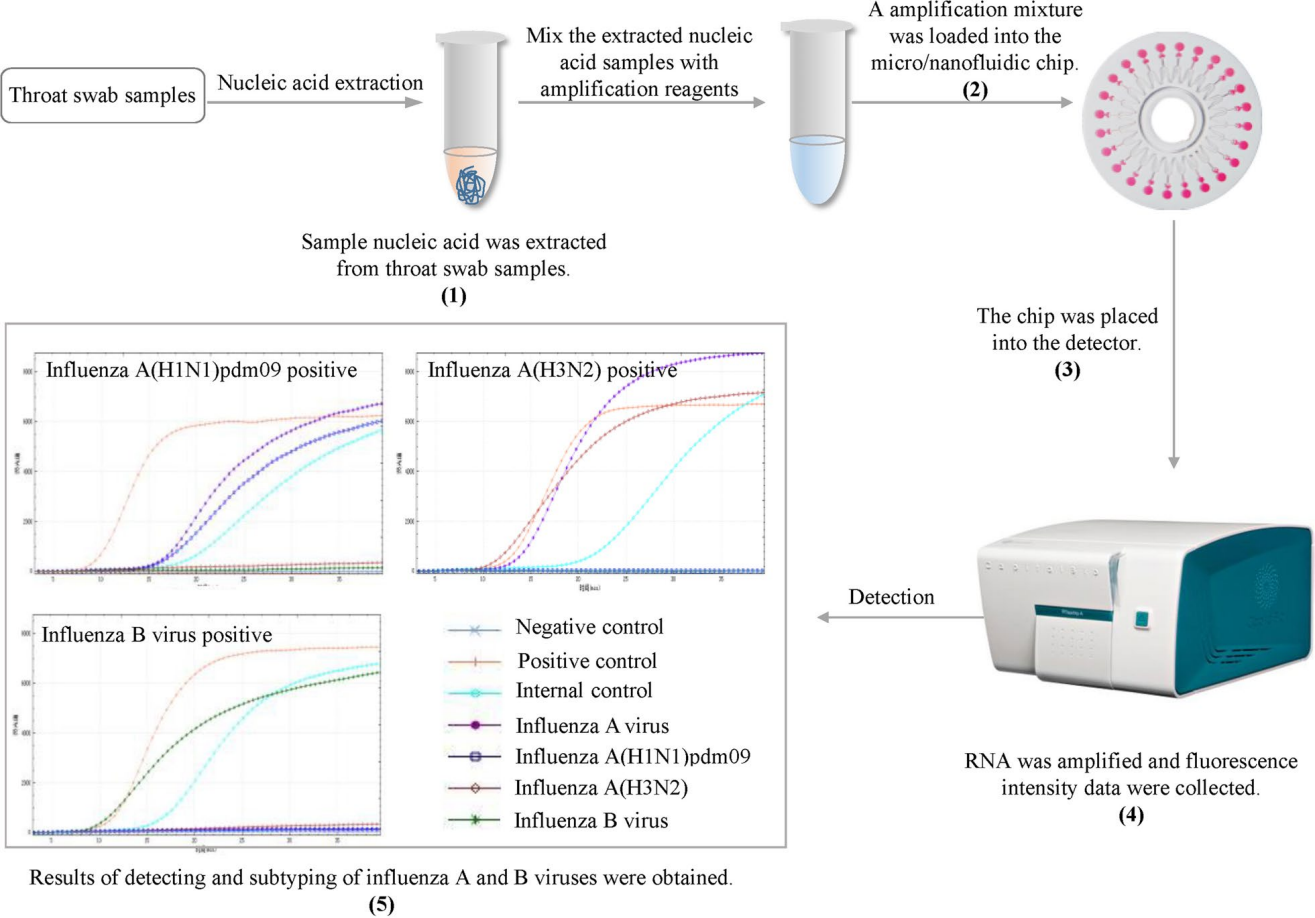
The workflow of MNCP. The steps were as follows: (1) Nucleic acid was extracted from throat swab samples. (2) A mixture including nucleic acid, basic NASBA reaction reagents, primers, and molecular beacon probes was loaded into the micro/nanofluidic chip. (3) The loaded chip was placed into the RTisochip-A detector (CapitalBio, Beijing, China). (4) RNA was amplified based on the NASBA and the fluorescence intensity data were collected and analyzed using the software. (5) Results of detecting and subtyping of influenza A and B viruses were obtained. Fluorescent curves were plotted by detection time (X-axis) against fluorescent intensity (Y-axis) for influenza A(H1N1)pdm09, A(H3N2), and influenza B virus, respectively. A batch of the MNCP tests took about 1 h. MNCP: micro/nanofluidic chip platform; NASBA: nucleic acid sequence-based amplification

The general workflow of the MNCP is shown in Fig. 3. Briefly, nucleic acids were extracted from throat swab samples using TIANamp Virus DNA/RNA Kit (Tiangen Biotech, Beijing, China). The nucleic acid (8 µl) was mixed with the amplification reagents, and the mixture was loaded into the micro/nanofluidic chip, in which 24 reaction chambers were each connected to a sine-shaped infusing channel by a micro-channel. Four pairs of primers and molecular beacon probes specific for influenza A virus matrix protein (MP) gene, influenza A(H1N1)pdm09 hemagglutinin (HA) gene, influenza A(H3N2) HA gene, and influenza B virus MP gene were employed to detect subtypes of influenza viruses. The primer and beacon probe sequences were designed according to Table 1. Subtype influenza A(H1N1)pdm09 or A(H3N2) was identified if the influenza A(H1N1)pdm09 HA gene or A(H3N2) HA gene was positive. Influenza A(not subtyped) was identified as influenza A MP gene positive plus A(H1N1)pdm09 HA gene and A(H3N2) HA gene negative. A positive control, a negative control, and an internal control were applied for each sample test as quality control. The positive control and the negative control were used to confirm that the chip was working correctly. Glyceraldehyde 3-phosphate dehydrogenase (GAPDH) gene was used as the internal control to monitor whether the extraction of nucleic acids from the swab samples was successful and detect whether the amplification process was valid. For each assay, the positive control, the negative control, the internal control, and the four pairs of primers and molecular beacon probes were preloaded into seven different reaction chambers, and the chip was fully enclosed in a cover to eliminate the risk of contamination (Zhang et al. 2018). Fluorescence intensity was measured twice every minute and the data were analyzed using the software that came with the RTisochip-A detector (CapitalBio, Beijing, China).

**Table 1:** The primer and beacon probe sequences

Subtypes of influenza viruses	Primer or probe	Sequence (5′-3′)	Length (bp)
Influenza A MP	Forward primer	CACTDGGCACGGTGAGCGTGAA	201
	Reverse primer	AATTCTAATACGACTCACTATAGGGAGATTCTAACCGAGGTCGAAACG	
	Beacon probe	FAM-CCCGTCTTTAGCCAYTCCATGAGAGCCTCACGGG-BHQ1	
Influenza A(H1N1)pdm09 HA	Forward primer	ATGATAATACCAGATCCAGCA	173
	Reverse primer	AATTCTAATACGACTCACTATAGGGAGAGTTCAAGCCGGAAATAGCAAT	
	Beacon probe	FAM-CCCGTAATGCATATCTCGGTACCACTAGATTTCACGGG-BHQ1	
Influenza A(H3N2) HA	Forward primer	CATAAGGGTAACAGTTGCT	188
	Reverse primer	AATTCTAATACGACTCACTATAGGGAGAATGCTACTGAGCTGGTTCA	
	Beacon probe	FAM-CGCGTTTGAGGGTCTCCCAATAGAGCATCACGCG	
Influenza B MP	Forward primer	CCAAAACTGTTTCACCCATT	168
	Reverse primer	AATTCTAATACGACTCACTATAGGGAGAACAATAAACAGAGAGGTATC	
	Beacon probe	FAM-CCCGTTCAATACCTCCATGTTGTCAGAGAGTACGGG-BHQ1	

Four pairs of primers and molecular beacon probes specific for influenza A virus matrix protein (MP) gene, influenza A(H1N1)pdm09 hemagglutinin (HA) gene, influenza A(H3N2) HA gene, and influenza B virus MP gene were employed to detect four subtypes of influenza viruses

### rRT-PCR

Briefly, nucleic acid extracted from the swabs was tested by rRT-PCR using a commercial kit (Bioperfectus Technologies, Jiangsu, China) accredited by China Food and Drug Administration on the Roche Cobas Z480 real-time PCR system (Roche Diagnostics, Shanghai, China) and following the manufacturer’s instructions (Sun et al. 2013). The threshold cycle value of ≤ 34.7 and ≤ 34.8 was interpreted as positive for influenza A virus and influenza B virus, and the threshold cycle value > 34.7 and > 34.8 was interpreted as negative for influenza A virus and influenza B virus, respectively. A batch of rRT-PCR tests took about 2 h to complete. Viral concentration of the samples was calculated by comparing rRT-PCR results with the standard curves generated from inactivated standard influenza A and B virus following the manufacturer’s instructions. Ten influenza A virus positive samples and ten influenza B virus positive samples were randomly selected for HA gene sequencing (Beijing Genomics Institute of China) to validate the accuracy of the rRT-PCR method.

### Rapid influenza diagnostic test (RIDT)

Rapid influenza diagnostic test was performed using Clearview Exact Influenza A&B (ABON Biopharm Co., Ltd, Hangzhou, China) following the manufacturer’s instructions. The test strip was coated with antibodies against nucleoproteins of influenza A and B virus at different positions to distinguish antigens between influenza A and B virus (Yao et al. 2019).

### Statistical analysis

Results of MNCP were compared with results of rRT-PCR and RIDT respectively, and rRT-PCR was considered the standard. Qualitative variables were summarized by their frequency distribution. The sensitivity, specificity, positive predictive values (PPVs), negative predictive values (NPVs), overall concordance rate, the kappa value, and the 95% confidence intervals of MNCP and RIDT were determined. McNemar’s test and the kappa value were used to analyze the statistical difference and agreement between methods. The kappa value of 0.21–0.39, 0.60–0.79, and 0.90–0.99 indicated a minimal, moderate and almost perfect agreement between two methods, respectively (McHugh 2012). A *P*-value of < 0.05 was considered statistically significant. Statistical analyses were performed using SPSS 24.0 software (IBM SPSS, Chicago, IL, USA).

## Results

### Baseline characteristics and the results of rRT-PCR

The baseline characteristics of the patients were shown in Table 2. There were 48.50% (129/266) male and 51.5% (137/266) female, with a median age of 3.83 years (range from 1 month to 66.5 years). Most of the patients were from the pediatric department. RIDT, MNCP, and the rRT-PCR tests were conducted successively in 255 of 266 patients for influenza A virus detection (11 patients were excluded because of sample volume shortage), with a positive rate of 35.29% (90/255) by rRT-PCR. Influenza B virus detection was successful in 259 of 266 patients (seven excluded because of sample volume shortage), with a positive rate of 38.22% (99/259) by rRT-PCR. Results of rRT-PCR were validated by influenza virus HA gene sequencing of randomly selected 10 samples as the two methods were 100% in agreement.

**Table 2:** Baseline characteristics of the patients with influenza-like illness

Baseline characteristic	Patients (n = 266)
Age (years), median (range)	3.83 (0.08–66.5)
Distribution by age group (years), n (%)	
0–≤ 2	60 (22.56)
> 2–≤ 6	130 (48.87)
> 6–≤ 14	29 (10.90)
> 14–≤ 45	26 (9.77)
> 45	21 (7.89)
Sex, n (%)	
Males	129 (48.50)
Females	137 (51.50)
Site of management, n (%)	
Outpatients	220 (82.71)
Admitted to the hospital	26 (9.77)
rRT-PCR positive, n (%)	
Influenza A virus	90/255 (35.29)^a^
Influenza B virus	99/259 (38.22)^a^

MNCP: micro/nanofluidic chip platform; RIDT: rapid influenza diagnostic test; rRT-PCR: real-time reverse transcription PCR

^a^RIDT, MNCP, and rRT-PCR test was conducted successively in 255 of 266 patients for influenza A virus detection (11 patients were excluded because of sample volume shortage), and in 259 of 266 patients for influenza B virus detection (seven excluded because of sample volume shortage)

### Results of MNCP compared with rRT-PCR

The positive, negative, and internal controls of all samples tested showed expected results indicating that all MNCP results were valid. Compared with rRT-PCR, the sensitivity, specificity, PPVs, NPVs, and overall concordance rate of MNCP were 98.89%, 96.97%, 94.68%, 99.38% and 97.65% for influenza A virus, and 94.95%, 99.38%, 98.95%, 96.95% and 97.68% for influenza B virus, respectively. The positive concordance rate, negative concordance rate, and overall concordance rate of MNCP and rRT-PCR were 98.89% (89/90), 96.97% (94/99), and 97.65% (249/255) for influenza A virus detection; and 94.95% (160/165), 99.38% (159/160), and 97.68% (253/259) for influenza B virus detection, respectively. The methodological comparison results showed that there was no statistical difference between MNCP and rRT-PCR for influenza A and B virus detection (McNemar’s test, both *P *= 0.219). The kappa value between MNCP and rRT-PCR for influenza A and B virus detection was both 0.95, indicating a near perfect agreement of both methods (Table 3).

**Table 3:** Results of the MNCP assay for influenza A and B virus detection and compared with the rRT-PCR test

Virus	No. detected (MNCP/rRT-PCR)^a^				Sensitivity (%) (95% CI)	Specificity (%) (95% CI)	PPVs (%) (95% CI)	NPVs (%) (95% CI)	Overall concordance rate (%) (95% CI)	*P* value (McNemar’s test)	Kappa value (95% CI)
	+/+	+/−	−/+	−/−							
Influenza A	89	5	1	160	98.89 (93.10 − 99.94)	96.97 (92.70 − 98.88)	94.68 (87.45 − 98.03)	99.38 (96.07 − 99.97)	97.65 (94.70 − 99.04)	0.219	0.95** (0.91 − 0.99)
Influenza B	94	1	5	159	94.95 (88.06 − 98.13)	99.38 (96.04 − 99.97)	98.95 (93.44 − 99.95)	96.95 (92.65 − 98.87)	97.68 (94.78 − 99.05)	0.219	0.95** (0.91 − 0.99)

+: positive; −: negative; MNCP: micro/nanofluidic chip platform; rRT-PCR: real-time reverse transcription polymerase chain reaction; CI: confidence interval; PPV: positive predictive value; NPV: negative predictive value

**P < 0.001 (kappa statistics)

^a^MNCP and rRT-PCR were conducted successively in 255 of 266 patients to detect influenza A virus (11 were excluded because of sample volume shortage) and in 259 of 266 patients to detect influenza B virus (seven excluded because of sample volume shortage)

Six of 255 samples showed opposite results between MNCP and rRT-PCR for influenza A virus detection (1, MNCP negative and rRT-PCR positive; 5, MNCP positive and rRT-PCR negative). Six of 259 samples showed opposite results for influenza B virus detection (5, MNCP negative and rRT-PCR positive; 1, MNCP positive and rRT-PCR negative) (Table 4).

**Table 4:** Samples with opposite results between the MNCP assay and rRT-PCR test

Opposite results	No.	MNCP results (time of positive/minutes)							rRT-PCR results (cycle of threshold)	
		Positive control	Negative control	Internal control	Influenza A virus	A(H1N1)pdm09	A(H3N2)	Influenza B virus	Influenza A virus	Influenza B virus
Influenza A virus detection	1	+/8.5	−	+/16.1	−	−	−	−	+/34.6	−
	2	+/11.2	−	+/15.2	+/21.4	−	+/16.8	−	−/36.1^a^	−
	3	+/11.2	−	+/14.7	+/19.2	−	+/13.1	−	−/35.2^a^	−
	4	+/12.0	−	+/22.1	+/35.6	−	+/28.6	−	−/37.6^a^	−
	5	+/13.4	−	+/16.4	+/23.9	−	−	−	−/UNDET^a^	−
	6	+/9.5	−	+/12.6	+/19.6	−	−	−	−/UNDET^a^	−
Influenza B virus detection	1	+/10.5	−	+/13.6	−	−	−	+/16.1	−	−/36.3^a^
	2	+/7.9	−	+/14.4	−	−	−	−	−	+/34.8
	3	+/8.5	−	+/20.6	−	−	−	−	−	+/30.5
	4	+/6.9	−	+/13.9	−	−	−	−	−	+/34.3
	5	+/7.7	−	+/15.7	−	−	−	−	−	+/32.5
	6	+/14.7	−	+/18.2	−	−	−	−	−	+/32.3

+: positive; −: negative; MNCP: micro/nanofluidic chip platform; rRT-PCR: real-time reverse transcription polymerase chain reaction; UNDET: undetected

^a^According to the reagent instruction, cycle of threshold values of > 34.7/UNDET and > 34.8/UNDET in rRT-PCR tests were interpreted as negative for influenza A virus and influenza B virus, respectively

### Subtyping the influenza virus by MNCP

Infection of influenza virus genre and its subtyping could be achieved within 1 h with the MNCP assay using one chip for each sample. Results showed that influenza A(H1N1)pdm09, A(H3N2), A(not subtyped), and influenza B virus accounted for 27.84% (71/255), 7.06% (18/255), 1.96% (5/255), and 36.68% (95/259) respectively in the samples tested.

### Results of MNCP compared with RIDT

The methodological comparison results in Table 5 showed that the overall concordance rate between MNCP and RIDT for the detection of influenza A and B virus was 70.98% (181/255) and 83.78% (217/259), respectively. Seventy-four of 255 samples for influenza A virus detection and 42 of 259 samples for influenza B virus detection showed opposite results, showing significantly statistical difference between the two methods (McNemar’s test, *P* < 0.001). The kappa value was 0.25 for influenza A virus detection and 0.62 for influenza B virus detection. Samples with opposite results between MNCP and RIDT were further compared with rRT-PCR results. The MNCP and rRT-PCR results of these samples showed a good agreement for influenza A and B virus detection, with an overall concordance rate of 93.24% and 95.24%, sensitivity of 100.0% and 97.56%, PPVs of 93.24% and 97.56%, respectively. No statistical difference between the MNCP and rRT-PCR methods was found (McNemar’s test, *P* > 0.05) (Table 6).

**Table 5:** Results of the MNCP assay for influenza A and B virus detection and compared with RIDT

Virus	No. detected (MNCP/RIDT)^a^				Sensitivity (%) (95% CI)	Specificity (%) (95% CI)	PPVs (%) (95% CI)	NPVs (%) (95% CI)	Overall concordance rate (%) (95% CI)	*P* value (McNemar’s test)	Kappa value (95% CI)
	+/+	+/−	−/+	−/−							
Influenza A	20	74	0	161	100.00 (79.95–100.00)	68.51 (62.10–74.31)	21.28 (13.78–31.17)	100.00 (97.10–100.00)	70.98 (65.41–76.55	< 0.001	0.25** (0.11–0.40)
Influenza B	54	41	1	163	98.18 (89.00–99.91)	79.90 (73.61–85.04)	56.84 (46.29–66.84)	99.39 (96.14–99.97)	83.78 (79.29–88.27)	< 0.001	0.62** (0.51–0.72)

+: positive; −: negative; MNCP: micro/nanofluidic chip platform; RIDT: rapid influenza diagnostic test; CI: confidence interval; PPVs: positive predictive values; NPVs: negative predictive values

**P < 0.001 (kappa statistics)

^a^MNCP and RIDT were conducted successively in 255 of 266 patients to detect influenza A virus and in 259 of 266 patients to detect influenza B virus

**Table 6:** Samples with opposite results between MNCP and RIDT were further compared with rRT-PCR results

Virus	No. detected (MNCP/rRT-PCR)^a^				Sensitivity (%) (95% CI)	PPVs (%) (95% CI)	Overall concordance rate (%) (95% CI)	*P* value (McNemar’s test)
	+/+	+/−	−/+	−/−				
Influenza A	69	5	0	0	100.00 (93.43–100.00)	93.24 (84.27–97.49)	93.24 (84.27–97.49)	0.06
Influenza B	40	1	1	0	97.56 (85.60–99.87)	97.56 (85.60–99.87)	95.24 (82.58–99.17)	1.00

+: positive; −: negative; MNCP: micro/nanofluidic chip platform; rRT-PCR: real-time reverse transcription polymerase chain reaction; CI: confidence interval; PPVs: positive predictive values

^a^Of 266 samples, 74 and 42 samples had opposite results between MNCP and RIDT for influenza A and B virus detection, respectively

### Virus concentration determined the agreement between RIDT and MNCP

**Fig. 4:**
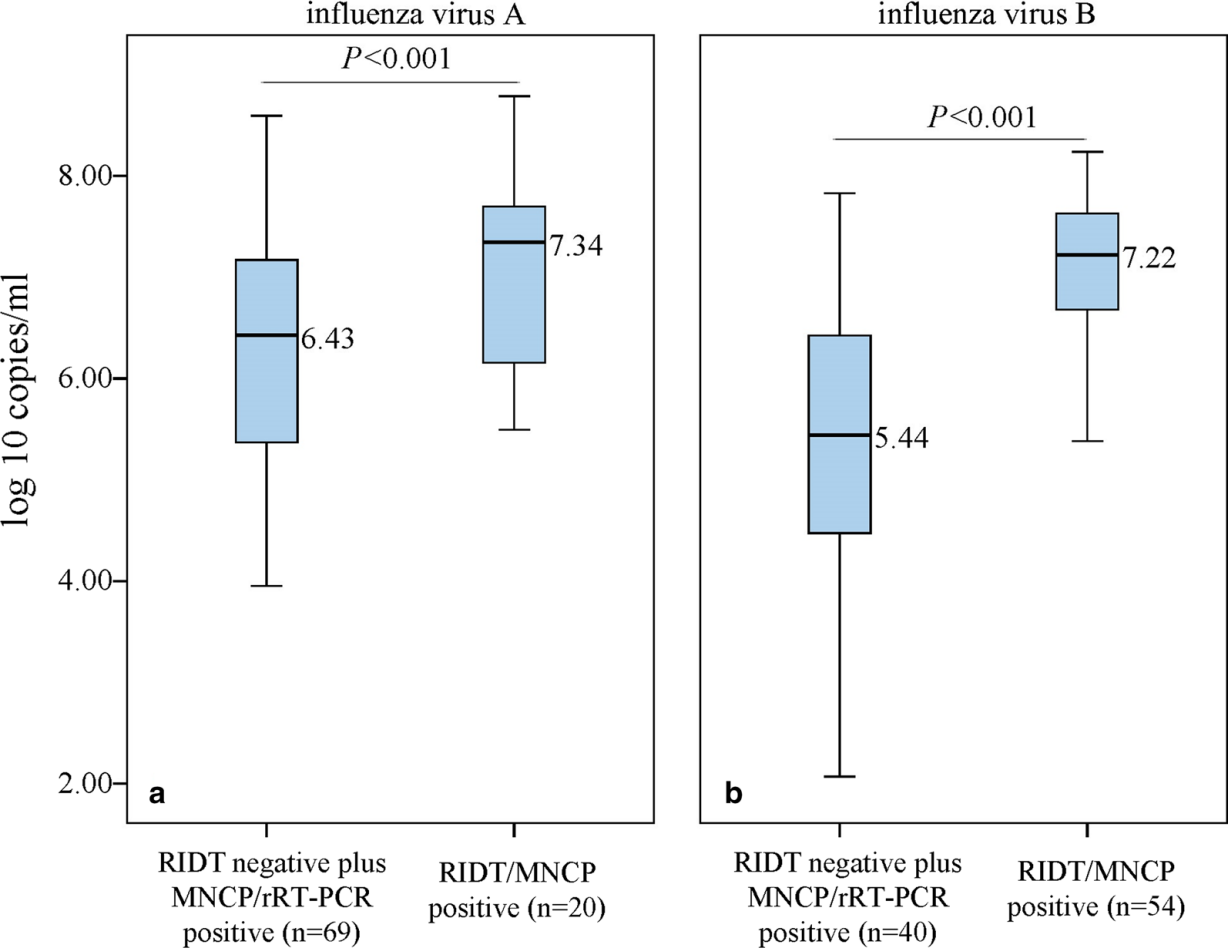
Box and whisker plots of the viral concentration distribution of samples with RIDT/MNCP positive results and RIDT negative and MNCP/rRT-PCR positive results determined by rRT-PCR (log 10 copies/ml). **a** Influenza A virus. **b** Influenza B virus. The box showed the median (thick line and number) and interquartile range (box length). The whiskers represented 1.5 times interquartile ranges or the highest/lowest points). MNCP: micro/nanofluidic chip platform; RIDT: rapid influenza diagnostic test; rRT-PCR: real-time reverse transcription PCR

As for influenza A virus detecting, 20 samples with RIDT/MNCP positive results had a median viral concentration of 2.19E+07 (10^7.34^) copies/ml, and 69 samples with RIDT negative plus MNCP/rRT-PCR positive results had a median viral concentration of 2.69E+06 (10^6.43^) copies/ml, indicating significantly statistical difference between the two groups (*P *< 0.001), as shown in Fig. 4a.

As for influenza B virus detecting, 54 samples with RIDT/MNCP positive results had a median viral concentration of 1.66E+07 (10^7.22^) copies/ml, and 40 samples with RIDT negative plus MNCP/rRT-PCR positive results had a median viral concentration of 2.75E+05 (10^5.44^) copies/ml, with significantly statistical difference between the two groups (*P *< 0.001), as shown in Fig. 4b.

## Discussion

The recently developed MNCP technique has been successfully used for rapid and high throughput detection of bacteria and viruses (Kaur et al. 2018), showing good sensitivity and specificity (Kim et al. 2017; Zhou QJ et al. 2014). Our study showed that compared with rRT-PCR, MNCP had good performance for influenza A and B virus detection, with the overall concordance rate, sensitivity, specificity, PPV, and NPV ≥ 95%, both the kappa values at 0.95, indicating almost perfect agreement with rRT-PCR. Subtypes of influenza A(H1N1)pdm09, A(H3N2), A(not subtyped), and influenza B virus could be distinguished simultaneously by MNCP within 1 h using one chip for each sample, which greatly reduced the workload and shortened the reporting time compared with rRT-PCR (2 h for one batch of test) (Lee et al. 2001). The amplification mixture of MNCP was loaded into different chambers of the chip, each chamber included one pair of primers and molecular beacon probes. It enabled the MNCP method to avoid the drawbacks of conventional multiplex PCR in which primer pairs significantly interfered and competed with each other in a single tube (Xu et al. 2015). In addition, because recombinant mutation of influenza A occurs frequently (Abed et al. 2005; Zhou J et al. 2014), influenza virus subtyping by MNCP will be a helpful tool for clinical and viral epidemiological studies, it can even recognize new recombinant mutants at an earlier stage.

Further analysis found opposite results between MNCP and rRT-PCR assays in small numbers of samples, six samples each in influenza A and B virus detection. According to the reagent instruction, cycle of negative threshold values for influenza A virus and influenza B virus were > 34.7/undetected, and > 34.8/undetected in rRT-PCR tests, respectively. Six samples with rRT-PCR positive/MNCP negative results for influenza virus B showed CT values were between 30.5 and 34.8. This study showed that MNCP may be more sensitive than rRT-PCR for influenza virus A detection, and less sensitive for influenza virus B detection (Xu et al. 2015; Zhang et al. 2018).

RIDT has been used clinically for a long time; however, the poor sensitivity of RIDT causes great concerns. This study also suggested low sensitivity of RIDT, since 74 of 255 samples for influenza A virus detection and 42 of 259 samples for influenza B virus detection showed opposite results between MNCP and RIDT. Using rRT-PCR as the standard method, MNCP results were in good agreement with rRT-PCR results, with an overall concordance rate of 93.24% and 95.24% for influenza A and B virus detecting, respectively. Considering that viral concentration might affect RIDT results, we quantitatively analyzed the virus concentration of the samples and the frequency distribution of the RIDT results. Samples with a median viral concentration of 2.69E+06 copies/ml for influenza A virus and 2.75E+05 copies/ml for influenza B virus could have RIDT negative results. The study further indicated that RIDT had a poor clinical sensitivity for influenza virus detection, which was in line with a report from Germany. In the German study, only 11% of 144 PCR-positive frozen clinical specimens were positive when tested retrospectively with Binax NOW Influenza A & B test (Inverness Medical), a commercial kit similar to the one used in our study (De la Tabla et al. 2010; Drexler et al. 2009). In consequence, a significant proportion of patients with false negative results in RIDT could lead to delayed diagnosis and treatment (Ginocchio et al. 2009). The single application of RIDT for the detection of influenza virus infection should be discouraged in favor of the clinical application of rRT-PCR and MNCP for patients with ILI (Drexler et al. 2009).

Our study also indicated that the application of RIDT to diagnose influenza A virus infection was more likely to have false negative results than to diagnose influenza B virus infection. This further suggested that the sensitivity of RIDT should be improved, especially for influenza A detection; as such testing results may mislead the clinical decision and epidemiological analysis. In recent years, the epidemic of influenza B virus has attracted much attention. Previous studies had suggested that influenza B virus could cause severe cases and fatalities as same as influenza A virus (Paul Glezen et al. 2013). It has been reported that the B/Yamagata lineage of influenza B virus was the predominant strain responsible for the 2017–2018 influenza epidemic in a global context after the B/Victoria lineage of influenza B virus, which caused global epidemic in the previous 2 years. Our study indicated that influenza virus B accounted for 38.22% of the cases, while 35.29% of the cases were caused by influenza virus A infection. Therefore, influenza B virus detection should be paid more attention to by clinicians.

For respiratory pathogen detection, poor quality specimens may induce false negative results for RT-PCR and RIDT (Drexler et al. 2009). It was worth noting that more concern should be given to the standardized sample collection. The swabs should be taken from the surface of the tonsils and the posterior pharyngeal wall, rather than from the surface of the oral cavity, to ensure that viruses can be collected (Eisfeld et al. 2014). In the MNCP detecting process, a positive control and a negative control were applied to monitor whether the chip was working correctly, while the internal control used the GAPDH gene to validate the swab sampling process. Using these controls could minimize the probability of having false-negative results that may mislead clinical decision.

This study had some limitations. (1) The MNCP chip was designed only for four subtypes of influenza virus. Therefore, other subtypes, such as influenza A(H1), A(H5), influenza B(Victoria) and B(Yamagata), were not included in the study. (2) The fact that MNCP reactions were in multiple chambers could increase the cost of running the assay. Ideally, both RNA extraction and amplification should be conducted using the same platform. (3) This was a single-center study and the sample population was relatively small.

In conclusion, MNCP was a homogeneous, isothermal amplification platform that can shorten the virus detection time for patients with ILI. The reaction chambers of the chip allow multiple influenza virus detection and subtyping. MNCP had the same sensitivity and specificity as rRT-PCR. MNCP was a novel, rapid, sensitive, and multi-purpose detecting system that has potential clinical values in pathogen diagnosis for patients with ILI.

## Data Availability

The data of this research are inserted in the present article; other data is available if needed.

## References

[CR1] Abed Y, Goyette N, Boivin G (2005). Generation and characterization of recombinant influenza A (H1N1) viruses harboring amantadine resistance mutations. Antimicrob Agents Chemother.

[CR2] Bedford T, Riley S, Barr IG, Broor S, Chadha M, Cox NJ, Daniels RS, Gunasekaran CP, Hurt AC, Kelso A, Klimov A, Lewis NS, Li X, McCauley JW, Odagiri T, Potdar V, Rambaut A, Shu Y, Skepner E, Smith DJ, Suchard MA, Tashiro M, Wang D, Xu X, Lemey P, Russell CA (2015). Global circulation patterns of seasonal influenza viruses vary with antigenic drift. Nature.

[CR3] Compton J (1991). Nucleic acid sequence-based amplification. Nature.

[CR4] De la Tabla VO, Antequera P, Masiá M, Ros P, Martin C, Gazquez G, Buñuel F, Sánchez V, Robledano C, Gutiérrez F (2010). Clinical evaluation of rapid point-of-care testing for detection of novel influenza A (H1N1) virus in a population-based study in Spain. Clin Microbiol Infect.

[CR5] Drexler JF, Helmer A, Kirberg H, Reber U, Panning M, Müller M, Höfling K, Matz B, Drosten C, Eis-Hübinger AM (2009). Poor clinical sensitivity of rapid antigen test for influenza A pandemic (H1N1) 2009 virus. Emerg Infect Dis.

[CR6] Eisfeld AJ, Neumann G, Kawaoka Y (2014). Influenza A virus isolation, culture and identification. Nat Protoc.

[CR7] Ginocchio CC, Zhang F, Manji R, Arora S, Bornfreund M, Falk L, Lotlikar M, Kowerska M, Becker G, Korologos D, de Geronimo M, Crawford JM (2009). Evaluation of multiple test methods for the detection of the novel 2009 influenza A (H1N1) during the New York City outbreak. J Clin Virol.

[CR8] Kaur A, Das R, Nigam MR, Elangovan R, Pandya D, Jha S, Kalyanasundaram D (2018). Rapid detection device for *Salmonella typhi* in milk, juice, water and calf serum. Indian J Microbiol.

[CR9] Kim S, De Jonghe J, Kulesa AB, Feldman D, Vatanen T, Bhattacharyya RP, Berdy B, Gomez J, Nolan J, Epstein S, Blainey PC (2017). High-throughput automated microfluidic sample preparation for accurate microbial genomics. Nat Commun.

[CR10] Lee MS, Chang PC, Shien JH, Cheng MC, Shieh HK (2001). Identification and subtyping of avian influenza viruses by reverse transcription-PCR. J Virol Methods.

[CR11] Li Q, Zhou L, Zhou M, Chen Z, Li F, Wu H, Xiang N, Chen E, Tang F, Wang D, Meng L, Hong Z, Tu W, Cao Y, Li L, Ding F, Liu B, Wang M, Xie R, Gao R, Li X, Bai T, Zou S, He J, Hu J, Xu Y, Chai C, Wang S, Gao Y, Jin L, Zhang Y, Luo H, Yu H, He J, Li Q, Wang X, Gao L, Pang X, Liu G, Yan Y, Yuan H, Shu Y, Yang W, Wang Y, Wu F, Uyeki TM, Feng Z (2014). Epidemiology of human infections with avian influenza A(H7N9) virus in China. N Engl J Med.

[CR12] McHugh ML (2012). Interrater reliability: the kappa statistic. Biochem Med.

[CR13] Neuzil KM, Wright PF, Mitchel EF, Griffin MR (2000). The burden of influenza illness in children with asthma and other chronic medical conditions. J Pediatr.

[CR14] Paul Glezen W, Schmier JK, Kuehn CM, Ryan KJ, Oxford J (2013). The burden of influenza B: a structured literature review. Am J Public Health.

[CR15] Rodríguez-Làzaro D, Lloyd J, Ikonomopoulos J, Pla M, Cook N (2004). Unexpected detection of DNA by nucleic acid sequence-based amplification technique. Mol Cell Probes.

[CR16] Sun Y, Jia T, Sun Y, Han Y, Wang L, Zhang R, Zhang K, Lin G, Xie J, Li J (2013). External quality assessment for Avian Influenza A (H7N9) virus detection using armored RNA. J Clin Microbiol.

[CR17] Townsend KA, Eiland LS (2006). Combating influenza with antiviral therapy in the pediatric population. Pharmacotherapy.

[CR18] Xu Y, Yan H, Zhang Y, Jiang K, Lu Y, Ren Y, Wang H, Wang S, Xing W (2015). A fully sealed plastic chip for multiplex PCR and its application in bacteria identification. Lab Chip.

[CR19] Yao Y, Zhipeng Z, Wenqi S, Runqing L, Dong Z, Kun Q, Xiuying Z (2019). Unreliable usage of a single influenza virus IgM antibody assay in influenza-like illness: a retrospective study of the 2016–2018 flu epidemic. PLoS ONE.

[CR20] Zhang G, Zheng G, Zhang Y, Ma R, Kang X (2018). Evaluation of a micro/nanofluidic chip platform for the high-throughput detection of bacteria and their antibiotic resistance genes in post-neurosurgical meningitis. Int J Infect Dis.

[CR21] Zhou J, Xu S, Ma J, Lei W, Liu K, Liu Q, Ren Y, Xue C, Cao Y (2014). Recombinant influenza A H3N2 viruses with mutations of HA transmembrane cysteines exhibited altered virological characteristics. Virus Genes.

[CR22] Zhou QJ, Wang L, Chen J, Wang RN, Shi YH, Li CH, Zhang DM, Yan XJ, Zhang YJ (2014). Development and evaluation of a real-time fluorogenic loop-mediated isothermal amplification assay integrated on a microfluidic disc chip (on-chip LAMP) for rapid and simultaneous detection of ten pathogenic bacteria in aquatic animals. J Microbiol Methods.

[CR23] Zhu D, Lok C, Chao S, Chen L, Li R, Zhao Z, Dong J, Qin K, Zhao X (2019). Detection and characterization of type B influenza virus from influenza-like illness cases during the 2017–2018 winter influenza season in Beijing, China. Arch Virol.

